# Digital Dermatology

**DOI:** 10.1111/ddg.70365x

**Published:** 2026-06-16

**Authors:** Lea Henkel, Avend Bamarni, Stephan Alexander Braun, Valentina Busik, Holger Andreas Haenssle, Stephan Rietz, Paul Schmidle, Sandra Schuh, Anna‐Theresa Seitz, Sebastian Sitaru, Stephan Traidl, Max Tischler, Felix von Krogh, Julia Welzel, Julia Winkler, Anastasia Sophie Vollmer, Katharina Susanne Kommoss, Alexander Zink

**Affiliations:** ^1^ Department of Dermatology and Allergy TUM School of Medicine and Health Technical University of Munich, Munich, Germany; ^2^ Department of Dermatology and Venereology, Medical Center ‐ University of Freiburg, Faculty of Medicine, University of Freiburg, Freiburg, Germany Medizinische Fakultät; ^3^ Christine Kühne‐Center for Allergy Research and Education (CK‐CARE) Davos Schweiz; ^4^ Department of Dermatology, Medical Faculty, University of Münster, Münster, Germany Universität Münster; ^5^ Department of Dermatology, Medical Faculty, Heinrich‐Heine University, Düsseldorf, Germany Heinrich‐Heine‐Universität; ^6^ Praxis für Dermatologie und Allergologie Dr. med. Thomas Führer Gießen Deutschland; ^7^ Department of Dermatology University medical Center Heidelberg Germany; ^8^ Department of Dermatology University medicine Mainz Mainz Germany; ^9^ Department of Dermatology and Allergology University Hospital Augsburg Augsburg Germany; ^10^ Department of Dermatology Venereology and Allergology University Hospital Leipzig Universitätsklinikum Leipzig AöR, Deutschland Leipzig Germany; ^11^ Department of Dermatology and Allergy Hannover Medical School Medizinische Hochschule Hannover Hannover Germany; ^12^ Facharzt für Dermatologie Haut+Laserpraxis Dr. Tischler+Team Dortmund Deutschland

**Keywords:** Artificial Intelligence, Big Data, Data security, Digitalization, Dermatology, Interoperability, Mobile applications, Patient‐centered care, Teledermatology

## Abstract

Digital transformation is fundamentally reshaping dermatology, creating new opportunities in diagnostics, therapy, and healthcare organization. Large datasets combined with Artificial Intelligence (AI) enable more precise classification and prognosis, particularly through the analysis of clinical and dermoscopic imaging. Increasingly, synthetically generated data is used to train new algorithms, though its clinical validity remains under evaluation. Teledermatology has established itself as an integral part of care. Video consultations and asynchronous image transfers enhance accessibility to dermatological expertise and can bridge care gaps, especially in structurally weak regions. Furthermore, mobile applications and digital platforms promote adherence, self‐monitoring, and active patient engagement. Despite these opportunities, challenges regarding data protection, interoperability, and regulatory frameworks must be addressed to ensure sustainable implementation. Interdisciplinary collaboration between medicine, technology, and health economics is crucial. Physicians play a central role as subject matter experts in assessing data quality and providing clinical interpretation of digital systems. Continuous training of medical staff remains essential. Overall, digitalization offers significant potential to improve dermatological care, provided there are structured processes, quality assurance, and consistent patient involvement.

## INTRODUCTION

Digitalization is no longer a topic for the future, but is already shaping dermatological care today. Hardly any other discipline relies so heavily on visual diagnostics, defined processes and interdisciplinary interfaces ‐ characteristics that make dermatology particularly predestined for digital innovations.^1^

From artificial intelligence (AI)‐supported image analysis through telemedical applications, electronic patient records (EPR), and big data approaches, as well as interactive tools such as wearables or avatars, new care models are emerging. These promote the active involvement of patients in prevention and therapy and open up prospects for more individualized and location‐independent care through continuous monitoring.

At the same time, digital transformation also brings challenges. Questions of validation, ethics, data protection and practical integration into everyday clinical practice must be answered before new technologies can develop their full potential. For doctors, this means building up digital skills in addition to traditional specialist knowledge and integrating them into everyday clinical routine.^2^

To provide expert guidance and support for these developments, various initiatives and networks have been established within the field of dermatology, including the Digital Dermatology Working Group within the German Dermatological Society (DDG), which promotes interdisciplinary exchange and the transfer of knowledge regarding digital applications.

This CME article provides an overview of different developments currently shaping the digital transformation in dermatology in Germany. It covers a wide range of topics, from the fundamentals of data usage and artificial intelligence to specific clinical applications, patient‐centered tools, as well as topics related to healthcare organizations. These not only reflect the current state of research and practice but also offer a glimpse into future developments.

## FUNDAMENTALS AND DATA BASIS

### Big Data

The term “Big Data” has increasingly gained prominence in the medical context in recent years. Originally coined by the IT analyst firm Gartner, it describes large volumes of data (Volume), high speeds of generation and processing (Velocity), and a great diversity of data (Variety) that cannot be efficiently processed using traditional methods. However, this definition is insufficient for the medical field, as data quality (veracity) and the potential benefit for patient care and research (value) also play a decisive role here.^3^

Big Data involves both structured and unstructured data from various sources: electronic health records, imaging data, molecular genetic profiles, patient‐generated health data, data from apps and wearables, and information from social media. This data can be collected, processed, analyzed, and incorporated into medical decision‐making processes as part of a data lifecycle.

Big Data is more than just a large volume of data—it describes a complex interplay of data collection, analysis, and application, with one of its goals being to improve medical care.

Big Data is more than just a large volume of data—it describes a complex interplay of data collection, analysis, and application, with one of its goals being to improve medical care.

It is also important to distinguish between data‐intensive applications and the use of machine learning. Not every big data application uses AI, and not every AI model requires large amounts of data.

Dermatology is particularly well‐suited for data‐driven applications. Visual diagnostics, chronic disease progression, and standardized scores provide a solid foundation for structured data collection. Registries such as TREATgermany or PsoBest enable the capture of real‐world clinical scenarios that yield important insights beyond those obtained through clinical trials. This includes, for example, data on treatment adherence, patient stratification, the long‐term benefits of biologics, or changes in quality of life during systemic therapy.^4^, ^5^ In addition, dermatological image data can be analyzed automatically. However, in practice, the use of big data presents significant challenges. A key prerequisite for reliable analyses is the structured and quality‐assured documentation of medical information. Even minor inconsistencies or a lack of standards can lead to erroneous conclusions. The basic principle of “garbage in, garbage out” (GIGO) illustrates this succinctly: Valid results can only be generated from high‐quality input data. ^6^

Big data is only as good as the underlying data—structure, standardization, and quality are essential for reliable results.

Big data is only as good as the underlying data—structure, standardization, and quality are essential for reliable results.

Another prerequisite for the sustainable use of medical data is adherence to the FAIR principles. These principles require that data be findable, accessible, interoperable, and reusable. In dermatological practice, this applies in particular to digital documentation, the semantic standardization of clinical parameters, and technical interoperability with other systems.^7^

Data protection also poses a major challenge. The processing of personal health data must comply with the General Data Protection Regulation. In this context, safeguarding individuals’ right to informational self‐determination is particularly important. Patients must be able to understand the purposes for which their data is being used. Transparent consent processes, clearly defined purposes, and technical security standards are essential.^8^

The use of algorithm‐based methods also carries the risk of systematic biases. Such biases can arise from unbalanced samples (sampling bias), incorrect label definitions (label bias), or unreliable input data (measurement bias). In dermatological image analysis, the insufficient representation of darker skin types is a particularly well‐known problem. Furthermore, many models, such as neural networks or support vector machines, offer little transparency to users, which can complicate clinical evaluation and reduce acceptance.

Amid these challenges, big data opens up a wide range of applications in dermatology: from automated diagnostics and personalized treatment support to population‐based prevention strategies. For this potential to be realized in clinical practice, clearly defined methodological, legal, and ethical frameworks are required, as well as interoperable IT structures, standardized documentation processes, and close collaboration between medical disciplines, data science, and healthcare decision‐makers.^3^, ^7^

A thorough understanding of digital technologies and data‐driven tools is increasingly becoming part of a physician's responsibilities. This requires continued development of expertise in handling health data in order to actively shape the quality of care as the digital transformation unfolds. However, the mere existence of these extensive data pools does not in itself generate any immediate clinical benefit. Extracting actionable knowledge from the raw data requires powerful analytical tools such as classification algorithms.

### Classification on a Large and Small Scale: Clinical and Laboratory AI in Dermatology

Big data provides the foundation for data‐driven applications, but its value only becomes apparent through appropriate analysis methods such as classification algorithms. In dermatology, these can support both laboratory processes and clinical applications.

AI classification algorithms have demonstrated significant diagnostic potential, particularly in distinguishing between malignant and benign skin tumors.^9^ Furthermore, AI‐based approaches also have potential applications in other areas of dermatology. For example, expert knowledge in the evaluation of native mycological samples, such as adhesive tape stripping, can be incorporated into AI algorithms.^10^

At the other end of the data spectrum are clinical images and full‐body scans.^11^ Here, AI can, among other things, enable automatic mapping to body regions,^12^ for example, to make unstructured image databases accessible to big data analyses. Once created, a dataset can also be used for future, typically more powerful algorithms.^10^

Dermatology offers ideal conditions for AI‐assisted classification: large amounts of imaging, dermatoscopic, and histopathological data are available and are relatively easy to standardize.

Dermatology offers ideal conditions for AI‐assisted classification: large amounts of imaging, dermatoscopic, and histopathological data are available and are relatively easy to standardize.

From a methodological perspective, it should be noted that classification algorithms are generally trained only for specific types of data and therefore produce valid results only when given appropriate input. The classification of (image) data represents a well‐established area of application for AI in medicine and specifically in dermatology, such as for distinguishing between benign and malignant lesions in dermatoscopic images.^9^ Current advances in computer science focus not only on large language models (LLMs) but also on algorithms for generating new (synthetic) image data,^13^ which could, amongst other things, be adapted for use in dermatology. Initial approaches to generating synthetic data – for example, to supplement datasets for classification algorithms – have already been successfully published in the computer science literature.^14^

While classification algorithms are primarily designed for the structured processing of specific data types, the next stage of development marks the transition to systems with a higher degree of autonomy, known as AI agents.

AI algorithms require specific data types and a high degree of standardization in their inputs to produce valid results.

### AI agents and agentic AI

Existing AI systems in dermatology are typically designed for clearly defined tasks and achieve high diagnostic accuracy, particularly in the interpretation of specific histological diagnoses.^15^ Since the end of 2022 at the latest, with the widespread availability of a popular so‐called large language model (LLM), a more general‐purpose AI has caught the attention of both physicians and patients. These programs are now also capable, to varying degrees, of processing, “understanding,” and even generating images, audio, and video material (generative AI model).

Current generative AI systems (e.g., ChatGPT, Claude, Gemini) require active user control via specific input commands, known as “prompts”.

Current generative AI systems (e.g., ChatGPT, Claude, Gemini) require active user control via specific input commands, known as “prompts”.

Automating such tasks could therefore represent the next step in their development. This requires a system capable of understanding a wide variety of heterogeneous data and processing it correctly.

In such a scenario, lab reports could be stored in a structured manner alongside other lab results, while the system immediately reports notable findings to the human user. At the same time, medical findings could be analyzed, potential inconsistencies with current data flagged for later review, and subsequently systematically archived.

AI agents are generative AI systems capable of making decisions and performing actions independently to achieve goals. Depending on the system's design, they can act alone (single‐agent) or work as a team (multi‐agent system).

All of these steps require an underlying generative AI model capable of processing images and language. However, a degree of autonomy is also required, and this is where AI agents come into play. An AI agent is a computer program based on an AI model that can interact independently with its environment to achieve a predefined or self‐defined goal.^16^

This requires the following basic components:

**Table:** 

A suitable AI model	AI model
Memory: the ability to recall sequences of events and the context of actions.	memory
Planning: breaking tasks down into subtasks or steps, prioritizing them, and coordinating the appropriate steps to reach a solution.	planning, reasoning
Tools: the ability to access data sources (such as emails, doctors’ notes, and current guidelines) and use data processing programs (such as MS Excel and practice management software).	tools
Self‐monitoring: Task progress and interim results must be monitored and, if necessary, adjusted when new findings warrant such changes.	state‐management

**FIGURE 1:**
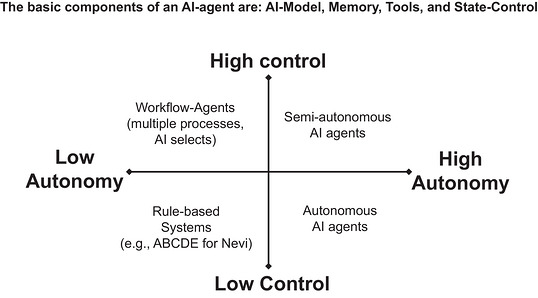
Types of AI agents depending on autonomy and human control. The graph illustrates the interplay between a system's independence (autonomy) and the degree of direct management by the user (control). While rule‐based systems exhibit low autonomy, autonomous AI agents operate largely independently with simultaneously reduced direct control.

In a medical setting, the ability to communicate and interact with human users (user interface, human‐in‐the‐loop) is desirable in order to monitor the agent's tasks and enable a meaningful collaboration. The key factor that enables a software program to act independently is the degree of autonomy it is permitted to exercise (Figure 1). A balance must be struck between autonomy and control.

Currently, there are no evidence‐based studies on the use of AI agents in dermatology. Agent‐based AI—that is, AI systems that interact autonomously with their environment to achieve predefined or self‐defined goals—may one day be used in scenarios such as the following:

- **Triage**: Automatic assessment of skin lesions, active follow‐up with patients, and requests for previous medical records. Synthesis of information, identification of urgent findings.
- **Monitoring**: Treatment monitoring through review of patient photos, reminders for follow‐up appointments, and necessary lab tests.
- **Semi‐autonomous therapy system**: Active monitoring of the therapeutic success of biologics for psoriasis using validated questionnaires, requesting progress photos from patients, maintaining a patient portal, and monitoring reported side effects. In the event of deviation from the treatment goal, recommending appropriate measures based on current guidelines.

Until agent‐based AI is widely adopted, it is essential to conduct high‐quality research to ensure that these agents benefit users, simplify work, and comply with legal requirements. Agent‐based AI systems intended for medical use are generally classified as medical devices under the EU MDR and are subject to a risk‐based conformity assessment.^17^ The EU AI Act classifies many AI systems in healthcare as high‐risk AI systems and requires, among other things, risk management, data and model governance, transparency, human oversight, and post‐market monitoring.^18^

When using digital technology—including AI agents—it is essential to first clearly define the problem before deciding on the feasibility, efficiency, and cost‐effectiveness of a system. It is equally crucial to understand the individual steps, processes, and communication interfaces involved in day‐to‐day operations in order to effectively integrate an AI agent into workflows to streamline tasks. Experience to date shows that analog workflows are often merely digitized (“electrified”) rather than being redesigned for the digital environment. For the successful integration of AI agents, fundamental process redesign (process re‐engineering) is a key success factor.^19^, ^20^

## CLINICAL APPLICATION OF AI

After presenting the basic concepts of big data and classification algorithms, we now turn our attention to their practical applications in dermatological practice. Imaging techniques in particular—such as dermatoscopy, LC‐OCT (Line‐Field Confocal Optical Coherence Tomography), or digital pathology—demonstrate how AI systems can support diagnostic processes, increase accuracy, and streamline workflows. At the same time, limitations are revealed that necessitate the responsible integration of these technologies into daily clinical practice.

### Performance and Limitations of AI‐Based Systems in Dermoscopy

Due to its focus on visual and morphological aspects, dermatology is particularly well‐suited for “vision‐based” AI systems.^21^, ^22^ Artificial neural networks (ANNs) have proven to be especially suitable for this area of “computer vision”.^23^, ^24^ After extensive training using clinical or dermoscopic images, ANNs are capable of indicating the probability of a specific diagnosis.^25^ This training corresponds to classical example‐based learning, or, in other words, “representation learning”.^23^ For the correct detection of malignant melanoma, this means that as many subtypes of melanoma as possible should be represented during training (i.e., nodular, acral, and amelanotic melanomas should be included).^26^ During training, an ANN must therefore learn, based on thousands of images, which morphological features characterize a melanoma and how these differ from the features of other skin tumors.^27^

Computer systems were already being used in skin cancer diagnosis prior to the introduction of ANN.^28^ These were based on simple feature recognition programmed by experts and demonstrated significantly lower diagnostic performance compared to today's ANN‐based systems.^29^ For the diagnosis of melanoma, the systems were programmed to detect asymmetry, number of colors, border characteristics, and lesion diameter. Understandably, this “rule‐based” approach could never capture every subtype or special form, ultimately resulting in a loss of diagnostic accuracy. A comparative study of a ANN‐based system and a “rule‐based” system revealed a striking difference, with the ANN‐based system demonstrating approximately 20% higher diagnostic performance.^29^ After initial feasibility studies demonstrated that ANN‐based systems could distinguish between melanomas and nevi with the same accuracy as trained dermatologists,^9^ in the following years research focused primarily on expanding training to include additional benign and malignant skin lesions. Today's competitive ANN‐based systems are typically trained to recognize the following diagnostic classes: melanoma (MEL), basal cell carcinoma (BCC), squamous cell carcinoma (SCC), actinic keratosis and Bowen's disease, melanocytic nevus (NV), dermatofibroma (DF), benign vascular tumors (VASC), and seborrheic keratosis and lentigo senilis. Even after expanding the training to include these additional classes, studies showed that the diagnostic accuracy remained on par with that of dermatologists.^30^ An initial prospective “real‐world” study on the collaboration between “man and machine” in melanoma diagnosis also provided compelling data demonstrating improved sensitivity and specificity through AI support.^31^

A comparative study of a ANN‐based system and a “rule‐based” system revealed a striking difference, with the ANN‐based system demonstrating approximately 20% higher diagnostic performance.^29^

**FIGURE 2:**
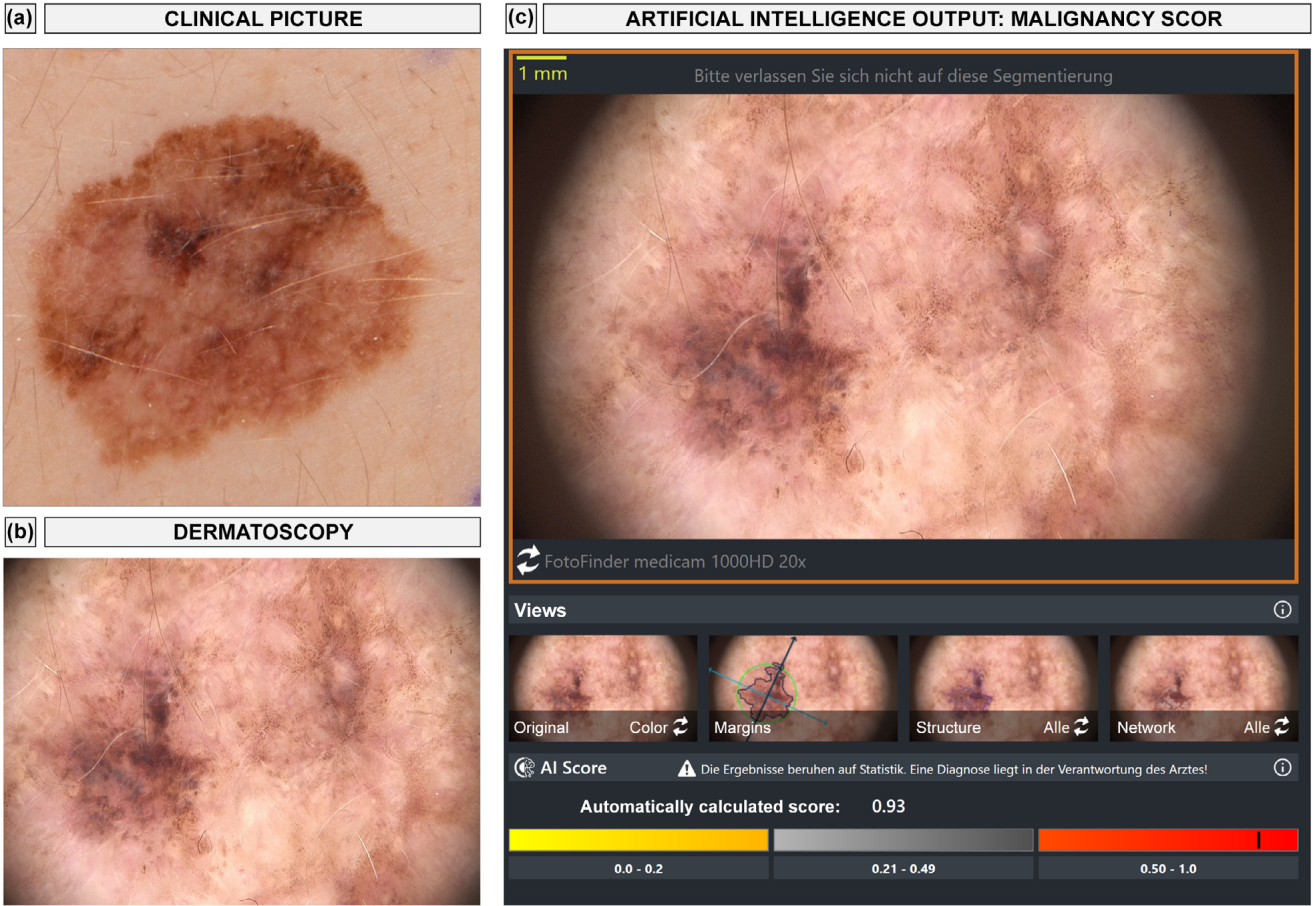
AI‐supported malignancy assessment of a superficial spreading melanoma. a) Clinical close‐up of an SSM with irregular borders and variegated pigmentation (tumor thickness 0.6 mm) on the back of a 53‐year‐old patient. b) Dermoscopy (20 x magnification, polarized light) showing signs of immunological regression and abundance of melanophages. c) The AI‐based output displays a malignancy score of 0.93 with a threshold for malignancy of ≥ 0,5 (FotoFinder Systems GmbH, Bad Birnbach).

Figure 2 illustrates the result of an AI analysis of a malignant melanoma. In this example, a probability of malignancy of 0.93 (i.e., 93 %) was reported (values ≥ 0.5 indicate a likely malignant lesion).

In general, dermatoscopic images appear to lead to correct diagnoses more frequently than clinical overview images or close‐ups of skin lesions, as more detailed (“feature‐rich”) images contain more diagnostically useful characteristics.^21^, ^32^

Furthermore, “expert systems” intended for use by physicians with appropriate medical certification must be distinguished from “lay systems” (mostly smartphone apps) intended for use by patients, in accordance with the Medical Devices Act. The comments above refer to expert systems, as the data available for “lay systems” can only be described as very unsatisfactory overall.^33^ Either the available study data indicate insufficient diagnostic performance, or such data are entirely lacking.

When using ANN‐based diagnostic systems in clinical practice, it is essential to be aware of their potential limitations and pitfalls. Neural networks with medical approval are assistive systems and provide only a single aspect of the information needed for a final diagnosis, which remains the responsibility of the physician. Furthermore, ANN‐based systems generally do not provide information on the degree of certainty or uncertainty with which a (correct or incorrect) diagnosis was made.^34^ Image artifacts such as colored markings on the skin can significantly impair the performance of ANN systems and should be strictly avoided.^35^ Another limitation arises from a lack of training images for rare diagnoses that fall outside the spectrum of the more common diagnostic classes mentioned above (for example, cutaneous lymphomas, rare adnexal tumors, cutaneous sarcomas, or rare subtypes of other tumors).

In summary, it can be concluded that AI‐based diagnostic systems for skin cancer diagnosis have established themselves in dermatology. Further prospective studies are needed to better investigate the benefits of such systems and explore additional areas of application. While dermatoscopy represents the most established application for AI, new non‐invasive imaging techniques such as LC‐OCT open up additional diagnostic possibilities.

For reliable AI diagnostics, training must include as many clinical subtypes of a given entity as possible (e.g., nodular or amelanotic melanomas).

### Integration of AI into Non‐Invasive Imaging

The accurate diagnosis of skin conditions such as actinic keratosis (AK), basal cell carcinoma (BCC), and psoriasis, as well as their quantification and monitoring over time, often poses a challenge in clinical practice. Conventional methods such as clinical examination or dermatoscopy provide valuable insights but quickly reach their limits when dealing with unclear lesions. Biopsies remain the diagnostic gold standard, but they are invasive and potentially stressful for patients (especially at delicate sites). For these reasons, non‐invasive diagnostic procedures are becoming increasingly important.

Line‐field confocal optical coherence tomography (LC‐OCT) is an imaging technique that provides microscopy‐like, three‐dimensional real‐time images of the skin – without the need for tissue sampling.^36^, ^37^ When combined with AI, this produces objective, reproducible images that can be used for both initial diagnosis and monitoring of disease progression. AI algorithms detect characteristic features such as epidermal changes and atypical cell nuclei.^38^

LC‐OCT allows for the visualization of typical histological features such as hyperkeratosis, parakeratosis, and atypia in AK diagnostics without the need for tissue biopsy.^39^, ^40^ AI‐supported analyses, such as the PRO‐Score for assessing basal epidermal proliferation, allow for a more refined risk assessment and facilitate monitoring of treatment success with non‐invasive therapy options.^41^, ^42^

**FIGURE 3:**
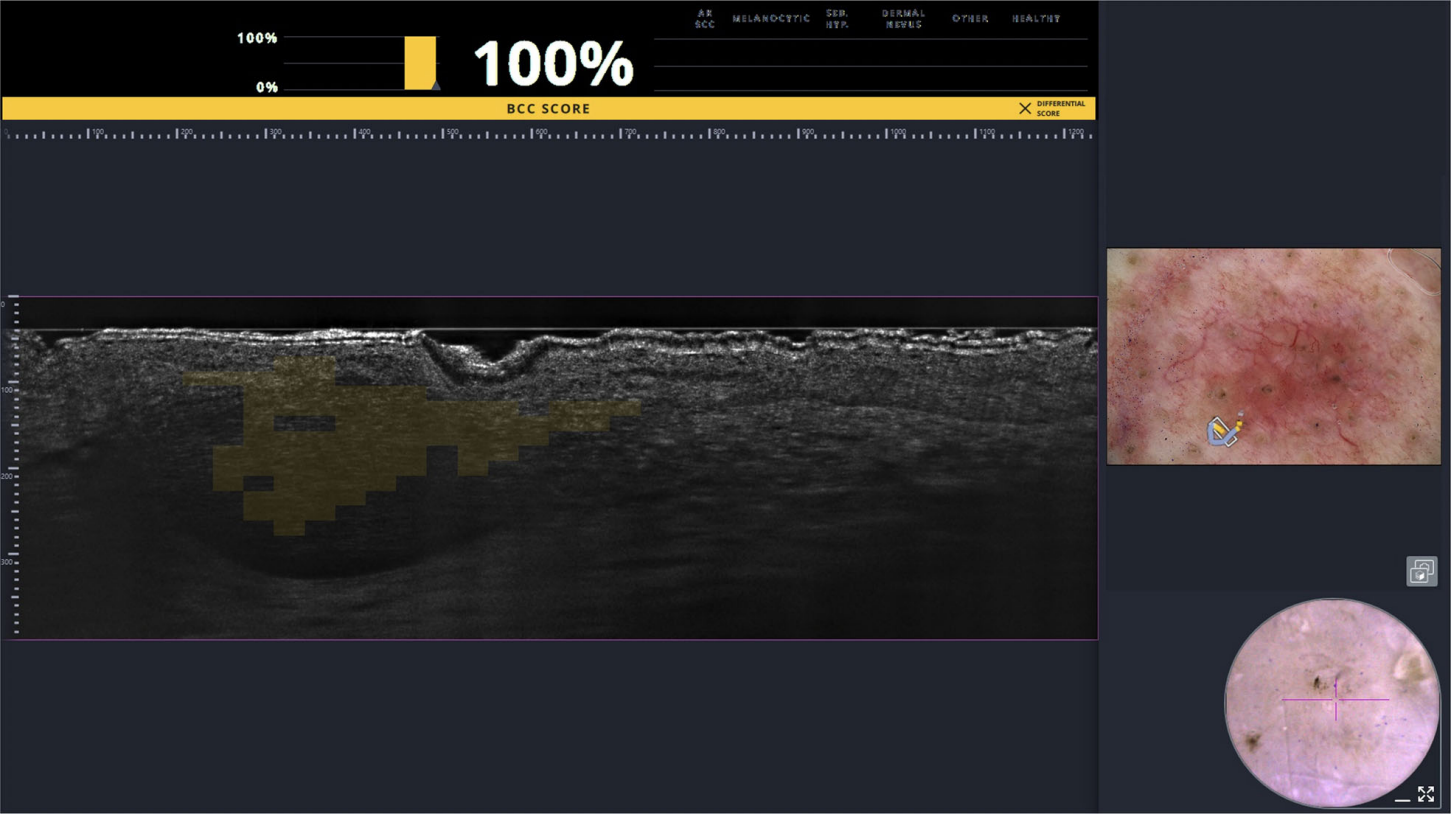
Line‐field confocal optical coherence tomography (LC‐OCT) of a nodular basal cell carcinoma (BCC). The figure displays both the LC‐OCT cross‐section and the AI‐generated overlay (heatmap), which highlights structures suspicious of tumor. A numerical "BCC Score" of 100 % indicates maximum probabi‐lity. Yellow marked segments designate areas with a very high probability (close to 100 %). Blue markings represent regions with a very low probability (close to 0 %). The color scale ranges from blue to yellow to red. Additionally, a clinical overview image (top right) and a dermoscopic image (bottom right) of the lesion are shown for better orientation.

Furthermore, there is significant benefit in BCC diagnostics: A deep learning algorithm trained on histologically annotated image data can distinguish between BCC and differential diagnoses in real time. Color‐coded heatmaps highlight suspicious areas directly in the image (Figure 3), which increases diagnostic confidence—especially among less experienced physicians.^43^ Multicenter studies demonstrate a significant improvement in sensitivity and specificity in the detection of BCC using LC‐OCT with AI support compared to clinical and dermatoscopic diagnostics alone.^43^ Furthermore, AI‐assisted margin marking of BCC can help precisely delineate tumors even before surgery.^44^, ^45^

Multicenter studies demonstrate a significant improvement in sensitivity and specificity in the detection of BCC using LC‐OCT with AI support compared to clinical and dermatoscopic diagnostics alone.^43^

LC‐OCT is a non‐invasive imaging technique that uses integrated, explainable AI to display real‐time heat maps in the images indicating the probability of a diagnosis.

AI‐assisted LC‐OCT also opens up new possibilities in psoriasis diagnostics: changes in epidermal and stratum corneum thickness can be measured with millimeter precision and documented throughout the course of treatment.^46^, ^47^ These objective parameters correlate closely with clinical response. While epidermal parameters often normalize quickly, vascular changes sometimes persist longer – a potential marker of persistent disease activity.^46^

However, integrating AI into daily clinical practice requires clear standards for image acquisition, data processing, and interpretation of results. Legal and ethical issues must also be addressed, particularly with regard to data protection and medical responsibility. AI should always be viewed as an assistive tool—the final authority for diagnosis remains with the physician.

The potential to reduce invasive procedures, speed up diagnoses and surgeries, and make dermatological care more personalized is groundbreaking. In addition to its practical relevance, this technology also opens up new avenues for research, such as the detailed analysis of tumor and inflammatory biology.^48^

While LC‐OCT complements routine diagnostic practice, digital workflows and AI are increasingly transforming histopathological diagnostics in the laboratory as well.

### Digitalization in Dermatopathology

Due to rapid technological advancements, digitalization is increasingly finding its way into dermatopathology as well, and on multiple levels.

On the one hand, organizational processes can be made significantly more efficient through a digital workflow that encompasses all laboratory steps, from sample acquisition and laboratory management to the digital transmission and archiving of findings.^49^, ^50^, ^51^

So‐called whole‐slide image (WSI) scanners make it possible to digitize entire histological sections easily, quickly, and with high quality, opening up entirely new ways of evaluating sections. The digitized glass slides are displayed in specialized image management systems. These systems not only allow for the simultaneous viewing of different stains and comparison with clinical images or other information, but also enable the rapid annotation of interesting structures within the tissue section. This makes it easier to retrieve them later for reviews or discussions. Additionally, WSI digital images can be more readily shared and discussed with experts worldwide.^52^

So‐called whole‐slide image (WSI) scanners make it possible to digitize entire histological sections easily, quickly, and with high quality, opening up entirely new ways of evaluating sections.

Furthermore, the spatial independence brought about by digitalization enhances the appeal of the profession, as flexible work arrangements (e.g., working from home) are now feasible.^53^ Given the declining number of practicing pathologists,^54^ and the steadily growing volume of specimens, finding ways to inspire dedicated young professionals to pursue a career in pathology is more important now than ever before.

Today, training in dermatopathology can also be conducted online without issue and regardless of location. The Münster histology course in its new digital format,^55^ and the online platform of the Working Group on Dermatological Histology (*Arbeitsgemeinschaft Dermatologische Histologie, ADH*), with its digital section seminars that have been successful for years, are prime examples thereof.^56^

Digital tissue sections form the basis for implementation of AI, which has also been the subject of increasing research and development in dermatopathology in recent years. A key focus is on AI‐assisted systems designed to support pathologists in time‐consuming routine tasks. Studies have already demonstrated their potential, for example in the detection of fungal elements in nail material,^57^, ^58^ or the automated detection, subtyping, and measurement of tumor thickness in basal cell carcinomas.^59^ The first of these systems, such as those for Ki‐67, Her2, ER/PR, or PD‐L1, are already CE‐certified in the EU and can thus be used in routine diagnostic procedures.

Vision‐language models are a new development that combines image and text information and is used as an advanced assistance system. In initial studies, they have already demonstrated the ability to respond to questions about histopathological sections,^60^ or to automatically generate diagnostics reports, for instance for basal cell carcinoma.^61^

In addition, current research into how AI can identify subvisual tissue features that could serve as digital biomarkers is underway.^62^, ^63^, ^64^ However, these methods are not yet part of routine practice.

AI‐assisted systems can support pathologists with time‐consuming routine tasks, such as the detection of fungal elements or the automated measurement of tumor thickness in basal cell carcinomas.

Nevertheless, there are factors that oppose the rapid, widespread digitization of dermatopathology. In addition to high acquisition costs for hardware and software, as well as the significant demand for storage capacity and energy, the primary challenge is the considerable amount of time required to restructure laboratory systems that have evolved over many years.^65^, ^66^ With regard to AI algorithms, there is also the question of diagnostic quality, for which excellent data quality is essential during development, as well as the inevitable legal and ethical issues associated with their use.

Many of these hurdles appear surmountable, suggesting that the future of dermatopathology will indeed be digital.

## DIGITAL TOOLS AND PATIENT‐CENTERED CARE

While the examples discussed so far have focused primarily on diagnostics and laboratory‐based applications, the next step will see patient‐centered digital tools gain particular importance. Technologies such as wearables (body‐worn digital monitoring systems), avatars (digital, interactive interfaces for patient communication), or digital communication platforms are shifting the focus from pure diagnostics toward the active involvement of patients in prevention, therapy, and care processes.

### Wearables

A prime example of patient‐centered digital technologies is wearables, i.e. electronic devices worn close to the body that continuously collect health‐related data and actively involve patients in prevention and disease management.

Wearables (e.g., smartwatches) and “smart‐skin” sensors are evolving from lifestyle gadgets into clinical tools. Miniaturized, skin‐worn systems continuously record parameters such as temperature, humidity, pressure, pH, or transepidermal water loss and, depending on the sensor technology, can even detect biomarkers in sweat (electrolytes, metabolites, proteins, hormones).^67^ For dermatology, this opens up new options for monitoring and personalized care; longitudinally collected data make it possible to map triggers, disease dynamics, and treatment effects in a way that reflects real‐life conditions.^68^, ^69^ In inflammatory skin conditions such as atopic dermatitis (AD), the focus is on predicting exacerbations. A wearable sensor currently under development (wristband form factor) combines classic skin parameters with electrodermal activity (EDA) as a surrogate for sympatheticotonic stress levels which can be a trigger that is often difficult to identify retrospectively. In addition, membrane‐based sweat sensors have been described that measure cortisol non‐invasively; such stress markers could be linked to clinical scores and patient‐reported outcomes (PROs) to define personalized warning thresholds (“digital biomarkers”). A recent example of the added value of continuous, real‐world biosignals comes from a recently published study on multimodal wearable data during pregnancy: temperature, heart rate, and activity trends derived from ring sensors traced high‐resolution trajectories from conception through the postpartum phase and identified physiological deviations in cases of early pregnancy loss. This work underscores the feasibility and clinical relevance of high‐frequency, real‐world time series – a concept that can be applied to long‐term dermatological courses (e.g., flare prediction, therapy titration).^70^ Existing limitations include higher barriers to entry for older patients, a general reliance on technology and connectivity, and the significant need for integration into existing workflows. It is therefore crucial to integrate these systems with mHealth apps as an interface between the patient, the device, and the practice or clinic – not as a replacement, but as a complement to medical care. In practice, a step‐by‐step approach is recommended, beginning with the definition of a clear use case (e.g., AD flare monitoring) and relevant endpoints (PROs, exacerbation rate). This is followed by addressing data protection issues and obtaining consent, training the team, and finally evaluating the system in routine practice. Thus, the added value of digital dermatology can be achieved in a quantifiable and patient‐centered manner.^71^

Wearables (e.g., smartwatches) and “smart‐skin” sensors are evolving from lifestyle gadgets into clinical tools.

While wearables primarily provide data for monitoring, digital avatars represent a new form of communication and patient education that complements the doctor‐patient conversation.

### Avatar‐Generated Patient Education

Patient education is a central medical duty and a prerequisite for trust, treatment adherence, and informed decisions. In dermatology, this applies not only to major procedures such as surgical interventions but also to minor procedures, systemic therapies, and chronic conditions. In everyday clinical practice, the conditions are often unfavorable: physicians work under significant time pressure, while patients may be emotionally overwhelmed following distressing diagnoses or may encounter language barriers.^72^

The use of digital avatars can create a safe space in which standardized, understandable, and multilingual educational content is provided with consistent quality, thereby complementing – but not replacing – the medical consultation.

The use of digital avatars can create a safe space in which standardized, understandable, and multilingual educational content is provided with consistent quality, thereby complementing – but not replacing – the medical consultation.

A medical avatar is an AI‐based, digitally generated representation of a healthcare professional or a medically approved communication model that realistically depicts voice, facial expressions, and gestures. The content of the videos is defined by the respective practice or clinic and is medically reviewed. Patients can access this information both on‐site and at home, watch it multiple times, and process it at their own pace, for example together with family members.

Technically, such systems are based on LLMs and multimodal AI applications that translate text into speech, facial expressions, and movements. Once created, an avatar can thus be used for a variety of standardized informational videos.^72^

One example is pre‐surgery counseling for skin cancer: The avatar explains the procedure, risks, and follow‐up care. Patients receive a QR code or link and can review the information again at home. For people with limited German language skills, it is crucial that the content is available in their native language – a foundation for genuine participation.^73^

Many patients feel overwhelmed by the flood of information: technical terms may be difficult to understand, and following a diagnosis, they often lack the emotional capacity for immediate processing of complex content. Avatars address this problem by using language accessible to laypeople, avoiding technical terms, and providing subtitles. Patients can watch the videos as often as they like without fear of disrupting the clinic's workflow by repeatedly asking questions.

Educational discussions often take longer than the actual examination—for example, during colonoscopies or gastroscopies. If patients do not understand the preparation instructions, the examination must be repeated. Avatars can prevent this by clearly conveying the instructions. At the same time, they reduce follow‐up inquiries to primary care physicians and medical assistants, thereby relieving pressure on the healthcare system. This allows physicians to focus more on individual inquiries rather than providing standard educational discussions under time pressure.^72^

A current example is the ArztAvatar.de project. The goal is to enable doctors to create a personalized avatar using their own face and voice in just a few minutes. Rather than replacing medical communication, this approach enhances it: patients enter the consultation better prepared, and doctors gain valuable time.^73^

The current focus is on non‐interactive formats. At Mainz University Medical Center, a clinical, questionnaire‐based study is underway involving patients undergoing surgery for skin cancer: The control group receives standard patient education, while the intervention group first receives avatar‐based education followed by a consultation with a physician. The goal is to examine understanding, acceptance, and time savings. In parallel, a medical history avatar is being developed to handle structured questioning and documentation.

In addition, the study examines how avatars can be used in medical education. To this end, virtual patients with different characteristics – such as being anxious, linguistically limited, or confrontational – are created to train medical students in handling critical counseling situations.

Avatars might not only help patients, but also serve as a training tool for future doctors.

Avatars might not only help patients, but also serve as a training tool for future doctors.

### Opportunities

**Table:** 

**Standardization**	Content is medically reviewed and available in consistent quality.
**Comprehensibility**	Clear language, avoidance of technical jargon, and subtitles significantly improve understanding.
**Trust**	By using the faces and voices of the treating physicians, the personal relationship is maintained.
**Patient Empowerment**	Information can be paused, replayed, is easy for laypeople to understand, and is available in the patient's native language.
**Efficiency**	Physicians save time on repetitive, routine patient education and can focus more on individual questions.
**Scalability**	An avatar can generate hundreds of videos without any extra effort for the practice.

### Challenges

**Table:** 

**Legal**	According to Section 630e of the German Civil Code (BGB), in‐person consultations remain mandatory. Avatars may only be used as a supplement.
**Data Protection**	GDPR compliance, server location, and preventing uncontrolled model training using patient data are critical.
**Acceptance**	Older patients or those with little experience of digital technology often prefer in‐person contact and may find the use of video or tablets overwhelming.

In the coming years, avatars will evolve into interactive, empathetic, and adaptive systems. Integrated into telemedicine platforms, hospital apps, or medical history stations, they can become an important part of patient care. Combined with AI‐supported triage systems or digital patient twins, the result is an ecosystem that reduces the burden on physicians while simultaneously improving patient communication.^72^, ^73^

The opportunity for a dual transformation presents itself: more time for the core medical task and greater clarity for patients.

The opportunity for a dual transformation presents itself: more time for the core medical task and greater clarity for patients.

However, alongside this structured, medically approved communication, unregulated information channels such as social media are also gaining increasing influence over health behaviors.

Avatar‐generated educational videos allow patients to process complex information at their own pace and in their native language – a prerequisite for making informed decisions.

### Social Media and Science Communication: Opportunities and Risks for Medical Care

Digital media are increasingly shaping social reality: Today, almost everyone has access to the internet, the average daily screen time in Germany exceeds five hours, and 77.6 % of the population actively uses social media (SM).^74^, ^75^ Statistically, people now spend more time in front of screens than in face‐to‐face conversations with friends or family. This development is having a profound impact not only on our social interactions but also on the role of the physician and the doctor‐patient relationship.^76^, ^77^

Medical knowledge is now available on an unprecedented scale. Information is freely accessible – through scientific blog posts, patient portals, online encyclopedias, podcasts, and platforms like PubMed – and is increasingly shared via social media. Here, TikTok, Instagram, YouTube, and streaming services such as Spotify and Apple Podcasts play a central role. Patients navigate an information landscape characterized by “information overload”.

Health‐related decisions are no longer made exclusively in doctors’ offices, but increasingly through discourse on social media platforms. Questions on lifestyle (such as “veganism”) or complex medical decisions (such as “cortisone anxiety” or biologic therapy) are discussed in public and controversially – often by laypeople, yet with a significant influence on individual health behaviors.

His is leading to a fundamental shift in the role of physicians: Today, physicians are less often mere conveyors of knowledge and more often experts in validating, interpreting, and contextualizing knowledge. This is leading to a fundamental shift in the role of physicians: Today, physicians are less often mere conveyors of knowledge and more often experts in validating, interpreting, and contextualizing knowledge. An increasing number of patients are already researching symptoms, diagnostic methods, and treatments before their consultation. In 2021, for example, 80 % of all cancer patients were already sharing information via social media.^78^

Today, physicians are less often mere conveyors of knowledge and more often experts in validating, interpreting, and contextualizing knowledge.

Questions, expectations, and treatment satisfaction increasingly depend on what content has been consumed beforehand via social media.^78^ Particularly problematic in this context is the spread of misinformation as well as the misuse of medical identities. Fake videos and deepfakes use the names and images of doctors without their knowledge to spread claims or promote products such as dietary supplements, often in violation of the German Law on the Advertising of Medicines (*Heilmittelwerbegesetz, HWG*).

In response, initial approaches to digital verification are emerging. Blockchain‐based authentication systems are intended to help users distinguish genuine content from counterfeits in the future. However, these methods are energy‐intensive and currently only scalable to a limited extent.^79^

For medical practice, this means that digital health literacy has become a key skill. Social media can be a valuable tool for conveying evidence‐based information in a patient‐centered manner. At the same time, this development requires active consideration of opportunities and risks, particularly with regard to misinformation, managing patient expectations, and integrating scientific evidence into public discourse.

The spread of misinformation and the misuse of medical identities through deepfakes on social media pose growing risks to patient safety and the physician‐patient relationship.

## HEALTHCARE AND ORGANIZATION

The digital tools described above illustrate how patients are increasingly becoming actively involved in their own care. However, for these innovations to reach their full potential, accompanying structural adjustments are needed in both private practices and hospitals. The following section therefore outlines how digital solutions—ranging from office organization to teledermatology—can be integrated into existing healthcare structures.

### Digital Practice Management in Dermatology

Dermatology has traditionally been considered an innovation‐friendly field, with digital procedures such as computer‐assisted videodermatoscopy having been part of routine clinical practice since long before the COVID‐19 pandemic.^81^ New digital laws, such as the Patient Data Protection Act (*Patientendaten‐Schutz‐Gesetz, PDSG*) and the Digital Act (*Digital‐Gesetz, DigiG*), have further reinforced this trend. Nevertheless, the widespread implementation of teledermatology in Germany remains incomplete to date. Although the pandemic theoretically acted as a catalyst, a nationwide study showed that over half of German dermatology clinics made little or no use of teledermatology applications during this period.^82^ This discrepancy demonstrates that technological availability alone is not enough to overcome the hurdles in standard clinical care.

### Digital Appointment Scheduling as an Organizational Foundation

Digital appointment scheduling systems have established themselves as a key tool for reducing the workload on practice teams. In addition to the basic booking function, they enable: automated appointment reminders via SMS or email, waitlist management for filling last‐minute openings, digital rescheduling in case of cancellations, and the ability to block patients with repeated unexcused no‐shows to reduce future missed appointments. This enables practices to achieve patient occupancy rates of 90% or higher, significantly reduce no‐shows,^80^, ^83^ and increase satisfaction among patients and staff.^84^ In addition, the booking process can be used to provide specific information about required documents, preparatory examinations, and wait times.

### Digital Medical History and Patient Education

Another key component of digitalization is the structured digital medical history. Patients fill out standardized questionnaires before their appointment via a web link or on a tablet at the practice. The collected information is promptly available in the practice management system via appropriate interfaces and can be flexibly adapted to individual parameters such as insurance status, reason for the appointment, or age.

Digital medical history tools increase efficiency by avoiding duplication and structured preparation of appointments.

Digital medical history tools increase efficiency by avoiding duplication and structured preparation of appointments.

### Reduced administrative burden through asynchronous communication (patient‐to‐physician)

In addition to appointment scheduling, routine inquiries can be effectively separated from regular practice activities using asynchronous teledermatology tools (“store‐and‐forward”). Studies show that 80–90% of these inquiries can be resolved purely through teledermatology, resulting in significant time savings for treatment processes.^85^, ^86^ The advantages include efficient triage and fully digital processing of trivial cases and progress monitoring.^87^ However, the current reimbursement situation remains a limiting factor: To date, there is no comprehensive billing code for asynchronous teledermatology in the Uniform Assessment Standard (*Einheitlichen Bewertungsmaßstab, EBM*) of the Statutory Health Insurance (*Gesetzlichen Krankenversicherung, GKV*), meaning that this service is often not reimbursable for SHI patients under standard care.

### Digital referral management through doctor‐to‐doctor consultations

An often underestimated means of relieving the workload on dermatology practices is the implementation of digital consultations between primary care physicians and specialists (“doctor‐to‐doctor”). Implemented via the Uniform Assessment Standard or regional structural agreements (for example, solutions such as Omnidoc^88^ or the pilot project “eDerm” of the German state of Saxony^89^), these systems serve as an efficient triage filter prior to the actual scheduling of appointments. Through the asynchronous assessment of general practitioners’ inquiries, unnecessary referrals can be avoided and urgent cases prioritized. An additional effect of this improved interface communication is a continuous transfer of knowledge, which sustainably strengthens the dermatological expertise of referring general practitioners.

### Online Reception as an Entry‐Level Solution

Online reception systems refer to portals that bundle various digital services such as appointment scheduling, prescription requests, or access to findings. Such solutions are particularly suitable for practices that have not yet achieved a high degree of digitalization, but they carry the risk of redundant processes and overlapping structures. Therefore, it is advisable to strategically select digital tools along a clearly defined patient journey.

### Electronic Medical Record (Elektronische Patientenakte, ePA)

The mandatory introduction of the electronic medical record (eHR) for all insured individuals creates new opportunities for cross‐sector care. Unless an objection is raised, medical findings can be viewed across institutions.^90^ Access for physicians is bound to an existing treatment context, which is typically authenticated by scanning the electronic health card.^91^ To ensure true interoperability, medical findings must be available in standardized formats such as Medical Information Objects (MIOs) so that they can be processed across different systems.^92^ The technical requirements for the practice include an up‐to‐date practice management system with an electronic medical record module and a connection to the telematics infrastructure.

### Shaping digital transformation together

The success of the digital transformation depends largely on the involvement of the entire practice team. Training, transparent communication, and the active involvement of medical assistants in digital processes increase acceptance and reduce anxiety.

Digitalization is not an end in itself – it lightens the workload when employees recognize the benefits it brings them.

Digitalization is not an end in itself – it lightens the workload when employees recognize the benefits it brings them.

Programs such as the “Digimanager Training” offered by the Westphalia‐Lippe Association of Statutory Health Insurance Physicians actively support practices and practice owners in the transformation process. Beyond practice organization, teledermatology opens up the possibility of location‐independent, flexible care.

Technological availability alone is not enough to overcome the barriers in routine clinical care; accompanying structural changes are needed in both medical practices and hospitals.

### Teledermatology

Teledermatology has become an essential component of dermatological care in recent years – driven by technological advances and the growing demand for dermatological services.^93^ The COVID‐19 pandemic marked a decisive turning point in teledermatological care. The extensive contact restrictions, which affected both the private and medical settings, led to a significant decline in dermatological care. This was offset by an expansion of telemedicine services to maintain dermatological patient care – at least in part.^94^ The importance of teledermatology is underscored by the existing German‐language S2k guideline,^95^ which highlights the established significance of this area of care and which is currently under revision.

In teledermatology, a primary distinction is made between synchronous and asynchronous methods; these are supplemented by combined procedures and hybrid models.

In teledermatology, a primary distinction is made between synchronous and asynchronous methods; these are supplemented by combined procedures and hybrid models.

In synchronous teledermatology, direct communication between patients and physicians takes place in real time, usually via a secure video connection. Live video consultations enable immediate medical history taking, visual assessment, and treatment recommendations. This offers particular advantages in outpatient care, such as reduced travel distances, expanded options for patients with limited mobility, more flexible scheduling, and a reduced risk of infection. At the same time, follow‐up questions can be addressed immediately. There are limitations when assessing discrete skin lesions, particularly those that require high‐resolution photographic documentation or dermatoscopic examination. Asynchronous teledermatology, on the other hand, is based on the store‐and‐forward principle. In this approach, clinical data and images are transmitted for evaluation by dermatologists at a later time. One example is the solution implemented at Leipzig University Hospital for in‐house teleconsultations. In this process, consulting physicians capture photos using a specialized app connected to the hospital information system. The dermatological assessment is then performed without direct patient contact, enabling flexible integration into daily clinical practice. In an evaluation of this method conducted between February and July 2023, 21.5 % (90 out of 419) of all dermatological consultations were handled via teledermatology.^96^ Dermatologists acting in a consultative capacity were able to fully resolve the issue via telemedicine in 92.7 % of these cases; nearly half also reported time savings of 30–60 minutes per consultation. In addition to reducing the burden on medical and nursing staff, patient transport and the associated risk of infection were also reduced.

An evaluation of clinical teleconsultations showed that over 90% of requests could be fully resolved via telemedicine, with time savings of up to 60 minutes per consultation.

Another innovative approach is the use of AI‐powered chatbots in teledermatology. These systems can conduct initial symptom assessments, collect structured medical history data, and formulate suspected diagnoses based on trained algorithms.

A well‐established example of this technology is Ada Health, which was founded in 2011 by Dr. Claire Novorol, Professor Martin Hirsch, and Daniel Nathrath. The app, which is still available in app stores today and has been downloaded over ten million times from the Google Play Store alone, underscores the high level of acceptance for such systems.^97^

This allows for targeted preparation of medical decision‐making processes and provides efficient support for case management. Particularly in conjunction with asynchronous teleconsultations, chatbots can help capture standardized image and diagnostics data, thereby improving the quality of dermatological assessments. A recent study by Shapiro et al. showed that a ChatGPT‐4‐based chatbot made the correct diagnosis in over 70 % of cases.^98^ Furthermore, in 84% of cases, it provided precise image descriptions with a quality that exceeded that made by physicians. This highlights the enormous potential of AI‐supported systems in future dermatological care.

Teledermatology can make a significant contribution to the quality of care in both outpatient and inpatient settings.

Teledermatology can make a significant contribution to the quality of care in both outpatient and inpatient settings.

**FIGURE 4:**
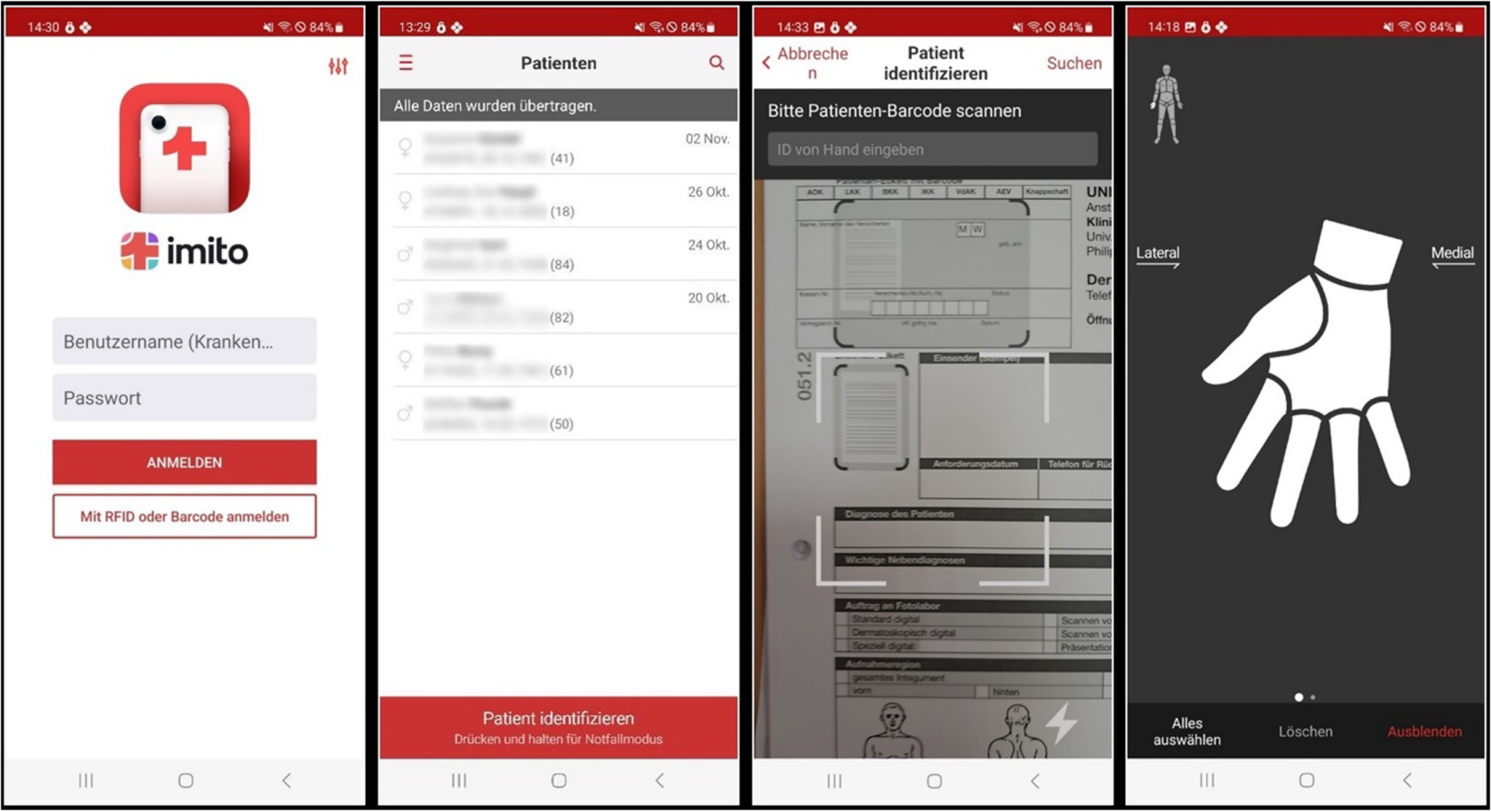
User interface of the imito app for clinical photo documentation. The sequence shows (f. L. t. r.): the login screen, the patient list from the connected Hospital Information System (HIS), the barcode scanning process for patient identification, and anatomical localization (body mapping) for precise marking of the affected skin area. The app facilitates a structured, asynchronous teledermatology workflow.

In the future, the combination of traditional teledermatology methods and supporting AI systems will offer further opportunities for an even more efficient and patient‐centered dermatological care (Figure 4).

### Virtual Patient Journeys in Dermatology

Dermatological care in Germany is currently organized primarily through in‐person visits and requires patients to navigate the healthcare system on their own. This includes choosing the first point of contact (primary care physician, specialist, hospital, or emergency room) as well as assessing the urgency of treatment. Given the limited number of dermatologists, specialist capacities are often overburdened by uncomplicated skin conditions such as warts, ringworm, or moles, while complex cases may not be adequately prioritized.

Virtual patient pathways integrate analog and digital care elements and provide structured approaches for improved patient management. Digital tools such as standardized medical history taking, symptom‐ and score‐based data collection, integration of wearables, image documentation, patient education, guidance on self‐management, and automated triage have so far been used only to a limited extent in dermatology.^99^, ^100^

The German Dermatological Society and the Working Group for Digital Dermatology (registered association, *Digitale Dermatologie e.V*.) are currently developing standardized and certified virtual patient pathways to supplement in‐person consultations. These are primarily intended for initial assessment (prioritization, referral) as well as for follow‐up care in cases of chronic skin conditions, wounds, postoperative recovery, oncological therapies, pruritus, and pain. Planned indication pathways include, among others, psoriasis, atopic dermatitis/hand eczema, prurigo nodularis/pruritus, acne inversa, urticaria, non‐surgically treated actinic keratoses/basal cell carcinomas, herpes zoster, dermatosurgical follow‐up care, and dermato‐oncological systemic therapies.

The concept envisages that patients receive access to an app via a barcode, through which they can answer disease‐related questions, record scores, upload images, and access disease‐specific information. Healthcare providers receive automated reports and alerts in the event of relevant changes, enabling continuous care rather than care provided only at set intervals.

In addition, medication reminders and information on complementary skin care help improve adherence and support prevention. The ability to use these pathways anytime and anywhere, along with cross‐sector collaboration among all participating healthcare providers, enables optimized resource utilization. Furthermore, the real‐world data collected through these pathways provides a valuable foundation for health services research.

Virtual patient pathways enable the automated classification, prioritization, management, and monitoring of skin conditions.

Virtual patient pathways enable the automated classification, prioritization, management, and monitoring of skin conditions.

In summary, virtual patient pathways offer the potential to further develop dermatological care in an evidence‐based, efficient, and patient‐centered manner.

## CONCLUSION AND OUTLOOK

Digitalization has already brought about lasting changes in dermatology and will continue to shape the field's development in the coming years. The examples described in this CME article demonstrate the diversity of digital innovations, ranging from data analysis and computer‐assisted diagnostic systems to patient‐oriented tools such as wearable monitoring devices and digital avatars, as well as new forms of practice organization, telemedicine consultations, and virtual patient pathways.

A common feature of these approaches is their potential for improving the quality of care, enhancing patient engagement, and providing targeted support for physicians’ work. At the same time, critical framework conditions, valid data, digital competencies, and responsible integration into daily clinical practice are essential.

Professional networks and platforms, such as the Digital Dermatology Working Group within the DDG, can help provide objective guidance for digital projects, link practical and research‐based experience, and promote practical implementation.

Overall, it is evident that dermatology can expand its diagnostics, therapeutic, and organizational potential through carefully considered and interdisciplinary integration of digital solutions. Innovations succeed when medical expertise, technical knowledge, and structured collaboration march hand in hand.

## CONFLICT OF INTEREST STATEMENT

H.A. Haenssle has received honoraria and/or travel expense reimbursements from companies involved in the development of skin cancer screening devices: Scibase AB, FotoFinder Systems GmbH, Heine Optotechnik GmbH, Magnosco GmbH.

M. Tischler is the medical director at OnlineDoctor 24 GmbH and a spokesperson for Doctolib.

L. Henkel, A. Bamarni, S.A. Braun, V. Busik, S. Rietz, P. Schmidle, S. Schuh, A. Seitz, S. Sitaru, S. Traidl, F. von Krogh, J. Welzel, J.K. Winkler, A.S. Vollmer, K.S. Kommoss and A. Zink have no conflicts of interest to declare.

## [[CME Questions / Lernerfolgskontrolle]]

1. Welche Aussage trifft auf KI‐Klassifikationsalgorithmen zu?
  - Ein Algorithmus kann grundsätzlich mit allen Datentypen (Bilder, Text, Audio etc.) umgehen, da alle Daten im Computer intern als Zahlen vorliegen.
  - Bei KI‐Klassifizierungsalgorithmen ist in nächster Zeit mit großen Performancesprüngen aufgrund neuer Modellarchitekturen zu rechnen.
  - Vor Anwendung in der Praxis muss man sich vergewissern, dass man den richtigen Datentyp für den Algorithmus wählt (z. B. klinisches oder dermatoskopisches Bild).
  - KI‐Algorithmen eignen sich NICHT, um strukturierte Daten z. B. das abgebildete Körperteil in klinischen Bildern aus großen Datenbanken zu erfassen.
  - Neue, durch KI generierte (=synthetische) Bilddaten eignen sich NICHT zum Trainieren neuer Algorithmen.
2. Worin liegt der praktische Nutzen von Heatmaps bei der KI‐gestützten BCC‐Diagnostik?
  - Sie erleichtern die Bestimmung der Eindringtiefe des Tumors.
  - Sie zeigen die Hautpigmentierung in unterschiedlichen Farbspektren.
  - Sie markieren Areale mit hoher Tumorwahrscheinlichkeit farblich.
  - Sie ermöglichen die Darstellung von Gefäßen in Echtzeit.
  - Sie reduzieren die benötigte Laserleistung des LC‐OCT‐Geräts.
3. Welche Aussage beschreibt am zutreffendsten den PRO‐Score in der LC‐OCT‐gestützten AK‐Diagnostik?
  - Er klassifiziert den Grad der dermalen Vaskularisierung.
  - Er bewertet die basale epidermale Proliferation in definierten Stufen.
  - Er misst die Dicke des Stratum corneum.
  - Er ordnet Läsionen nach Risikogruppen für BCC ein.
  - Er zeigt die Intensität des Entzündungsprozesses im Dermisbereich an.
4. Welche Aussagen treffen für die digitale Dermatopathologie nicht zu?
  - Erste KI‐Algorithmen sind bereits CE‐zertifiziert und können somit in der pathologischen Routinediagnostik eingesetzt werden.
  - Die Ausbildung der dermatopathologischen Nachwuchses wird zunehmend auch in den digitalen Raum verlagert.
  - Bestimmte Forschungszweige in der Dermatopathologie beschäftigen sich aktuell mit der Integration von visueller und Textinformation in sogenannten Language‐Vision‐Modellen.
  - Hohe Hard‐ und Softwarekosten beeinflussen die Geschwindigkeit der Digitalisierung in der Dermatopathologie in keiner Weise.
  - Durch zunehmende Digitalisierung in dermatopathologischen Laboren und Einführung eines digitalen Workflows kann auch auf organisatorischer Ebene die Effizienz deutlich gesteigert werden.
5. Welche rechtliche Grundlage ist trotz Avatar‐Aufklärung verbindlich?
  - DSGVO Art. 17 – Recht auf Löschung.
  - §630e BGB – Pflicht zum mündlichen Aufklärungsgespräch.
  - GOÄ §12 – Vergütungsregelung.
  - TMG §5 – Impressumspflicht.
  - Keine, da Avatare nicht geregelt sind.
6. Welche Aussage zu einer visuellen diagnostischen künstlichen Intelligenz (KI) trifft zu?
  - Eine visuelle KI lernt effektiv durch soziale Interaktion und Diskussion (social learning).
  - Der Diagnose einer visuellen KI kann immer vertraut werden, weil sie keine Fehler macht.
  - Eine visuelle KI zeigt oft eine geringere diagnostische Leistung für seltene Diagnosen.
  - Dermatoskopische Aufnahmen sind für eine visuelle KI grundsätzlich ungeeignet.
  - Viele Smartphone‐APPs (Laiensysteme) können heute schon einen Arztbesuch ersetzten.
7. Welche Aussage zur Teledermatologie trifft am ehesten zu?
  - Asynchrone Teledermatologie setzt ausschließlich auf Live‐Videogespräche und erlaubt keine zeitversetzte Beurteilung.
  - Es gibt bisher keine deutschsprachige Teledermatologie‐Leitlinie.
  - Teledermatologie ist aufgrund technischer Limitationen grundsätzlich nicht in der stationären Versorgung einsetzbar.
  - Synchrone Teledermatologie ermöglicht eine direkte Arzt‐Patienten‐Interaktion in Echtzeit, z. B. per Videoverbindung.
  - Teledermatologie hat keine Limitationen und ersetzt die klassische dermatologische Versorgung vollständig.
8. Welches primäre Ziel verfolgen die von der Deutschen Dermatologischen Gesellschaft (DDG) und Digitale Dermatologie e. V. entwickelten standardisierten virtuellen Patientenpfade?
  - Ersetzung sämtlicher Präsenzkontakte in der dermatologischen Versorgung.
  - Primärversorgung von Patientinnen und Patienten mit komplizierten Hauterkrankungen in der Notaufnahme.
  - Erstbeurteilung (Priorisierung, Zuweisung) und Verlaufskontrolle bestimmter dermatologischer Indikationen.
  - Entwicklung neuer medikamentöser Therapien für chronische Hauterkrankungen.
  - Etablierung einer flächendeckenden Hautkrebs‐Screeningpflicht.
9. Welches der folgenden digitalen Instrumente wird im Rahmen virtueller Patientenpfade nicht explizit genannt?
  - Standardisierte Anamneseerhebung.
  - Symptom‐ und scorebasierte Befunderfassung.
  - Integration von Wearables.
  - Virtuelle Realität zur Therapie chronischer Schmerzen.
  - Automatisierte Triage.
10. Welche zusätzliche Funktion der App im Rahmen der virtuellen Patientenpfade soll besonders die Therapietreue (Adhärenz) unterstützen?
  - Automatische Hautkrebsdiagnose mittels KI.
  - Erinnerung an Medikation und Anleitung zur Hautpflege.
  - Verpflichtende wöchentliche Videosprechstunde.
  - Bereitstellung von Laborwerten in Echtzeit.
  - Steuerung der Terminvergabe in dermatologischen Kliniken.

Liebe Leserinnen und Leser, der Einsendeschluss an die DDA für diese Ausgabe ist der 30. September 2026.

Die richtige Lösung zum Thema Die Kaltplasma‐Technologie in der Behandlung von Menschen mit chronischen Wunden in Heft 2/2026 ist: 1c, 2b, 3e, 4a, 5b, 6b, 7a, 8b, 9c, 10b

Bitte verwenden Sie für Ihre Einsendung das aktuelle Formblatt auf der folgenden Seite oder aber geben Sie Ihre Lösung online unter http://jddg.akademie‐dda. de ein.
